# A Rare Case of Transplanted Kidney Lymphangiectasia in a Patient With Joubert Syndrome

**DOI:** 10.7759/cureus.39814

**Published:** 2023-06-01

**Authors:** Mohamed Lameir Mukhtar Hussein, Jouhar J Kolleri, Sabir A Al Sharani, Amal M. J. Thabet, Akram Twair

**Affiliations:** 1 Clinical Imaging Department, Hamad Medical Corporation, Doha, QAT

**Keywords:** nm renogram, ultrasonography, magnetic resonance imaging, radiology, transplanted kideny, renal lymphangiectasia

## Abstract

Renal lymphangiectasia is one of the rarest surgical complications in post-transplant kidney patients. A few patients may clinically complain of nonspecific symptoms, and the other few might be diagnosed incidentally. We report the case of a 32-year-old female patient with a known case of Joubert syndrome who presented with nonspecific clinical manifestations. The patient underwent ultrasound, magnetic resonance imaging (MRI), and nuclear medicine (NM) imaging to confirm the diagnosis, which showed radiologic features of renal lymphangiectasia. Conservative medical management was delivered to the patient.

## Introduction

Renal lymphangiectasia is an uncommon benign abnormality that is manifested by distended peripelvic, intrarenal, and perirenal lymphatics. Its pathophysiology has been unclearly understood until now [1]. The clinical presentation of renal lymphangiectasia varies between asymptomatic, nonspecifically symptomatic, and renal insufficiency [2]. The diagnosis of renal lymphangiectasia is mainly dependent on imaging (ultrasonography, computed tomography, and MRI), and it is radiologically characterized by its cystic appearance [3]. Herein, we present a case of renal lymphangiectasia that was diagnosed by ultrasonography, MRI, and nuclear medicine (NM) imaging and managed conservatively.

## Case presentation

A 32-year-old female patient presented with mild right lower abdominal pain. There was no history of fever, dysuria, hematuria, or vomiting. She is a known case of Joubert syndrome and had a kidney transplant in 2005 from a living, unrelated donor with stable, normal graft function. On examination, she was vitally stable. A gastrointestinal examination showed a distended abdomen with a palpable, tender mass in the right lower abdomen. She was on a regular oral sirolimus 2 mg tablet daily.

**Figure 1:**
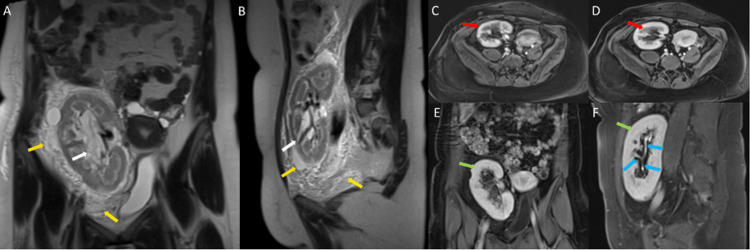
MRI of the abdomen with IV contrast: A) coronal T2W; B) sagittal T2W; and C) axial T1W fat saturated (fat sat) post-contrast arterial phase; D) axial T1W fat saturated post-contrast venous phase; E) coronal T1W fat sat post-contrast delayed phase; and F) sagittal T1W fat sat post-contrast delayed phase shows an enlarged transplanted kidney in the right iliac fossa with septated renal sinus cystic spaces (white arrows), perinephric fluid collection (orange arrows), and edema of the surrounding fat (yellow arrows). Post-contrast arterial and venous phases demonstrate adequate renal parenchymal enhancement (red arrows) and prompt excretion (green arrows) with opacification of the calyceal system (blue arrows) on delayed images. T2W: T2-weighted image; T1W: T1-weighted image

She had mild lower abdominal pain in 2014, and an MRI of the abdomen with intravenous contrast of the renal transplant was done. It showed an enlarged transplanted kidney in the right iliac fossa. It showed normal parenchymal enhancement with prompt excretion, a normal opacified non-dilated pelvic-calyceal system, dilated non-enhanced peripelvic cystic structures, and perinephric fluid with septations. These features were suggestive of peripelvic and perirenal cysts or lymphangiectasia of the renal transplant (Figure 1).

Her laboratory examinations were unremarkable, with slightly low albumin and no significant changes compared to her lab results in 2014, which indicated preserved renal functions (Table 1).

**Table 1:** Comparison between the lab parameters of 2014 and 2023 eGFR: estimated glomerular filtration rate.

Serum test parameter	2014	2023	Reference range
Urea	3.5	3.1	2.86-7.14 mmol/L
Creatinine	73	79	Female: 44.2-97.24 umol/L, Male: 61.9-114.9 umol/L
eGFR	>60	86	>60
Sodium	143	142	136-145 mEq/L
Potassium	3.6	4.0	3.5-5.0 mEq/L
Chloride	108	105	98-106 mEq/L
Bicarbonate	26.7	26	23-28 mEq/L
Calcium	2.02	2.27	2.2-2.7 mmol/L
Total protein	6.5	6.7	6.0-8.3 g/dL
Albumin	3.5	3.3	3.5-5.5 g/dL
Random blood glucose	4.0	4.2	3.9-5.6 mmol/L

**Figure 2:**
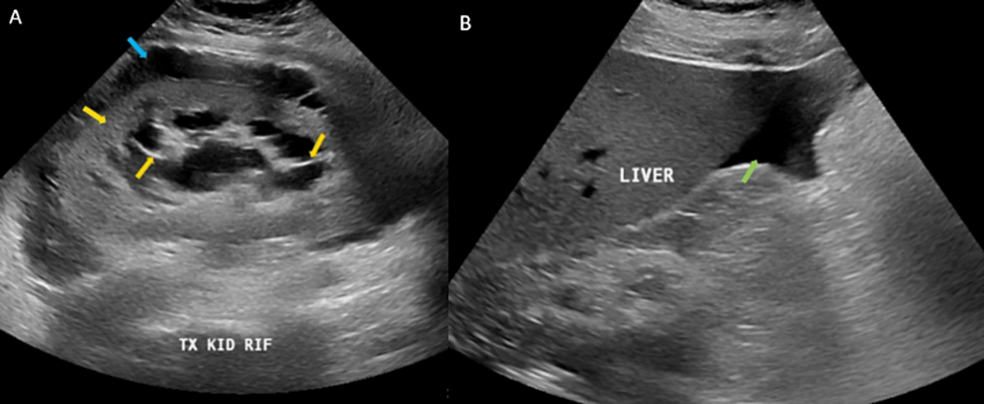
Gray-scale ultrasound-selected images of the transplanted kidney and upper abdomen show an increased parenchymal echogenicity of the kidney (yellow arrow), with renal peri pelvicalyceal cystic structures and hyperechoic wall/septae (orange arrows). There is a perinephric fluid collection with septae (blue arrow) along with minimal subhepatic free fluid (green arrow).

Ultrasound abdomen showed the transplanted kidney in the right iliac fossa, measuring 15.6 x 8.6 cm, with increased cortical echogenicity, a dilated pelvicalyceal system (the renal pelvis measured 18.4 mm), and septated perinephric fluid collection of approximately 263 ml in volume. Multiple cysts were noted, the largest measuring 4.3 x 4.4 cm. The resistive indices were 0.59, 0.58, and 0.67 in the upper, mid, and lower poles, respectively. Both native kidneys were echogenic and small. The right kidney measured 6.5 x 3.2 cm, and the left kidney measured 7 x 3.7 cm. No stones or hydronephrosis were seen. A trace of subhepatic fluid was noted (Figure 2).

**Figure 3:**
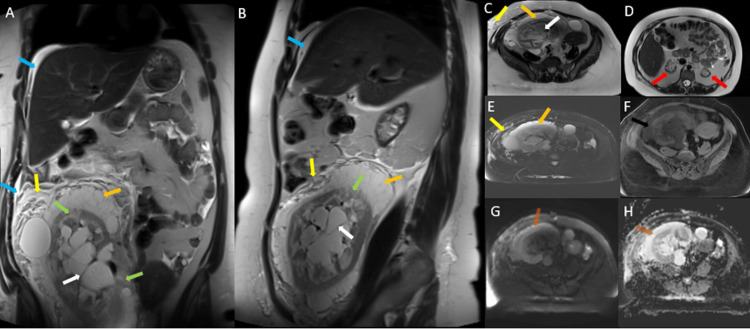
MRI of the abdomen: (A) coronal T2W; (B) sagittal T2W; (C & D) axial T2W; (E) T2W fat saturation (fat sat); (F) T1W fat sat; (G) DWI and (H) ADC images: showing an enlarged transplanted kidney in the right iliac fossa with linear cortical and peri-pyramidal high T2 signal (green arrows); septated peri pelvicalyceal cystic structures (white arrows); septated perinephric fluid (orange arrows) which appear hypointense on T1W fat sat (black arrow), with traces of fluid in the abdominal wall (yellow arrows); mild free fluid in the upper abdomen and pelvis (blue arrows) and no diffusion restriction (brown arrows). Native kidneys are atrophic (red arrows). T2W: T2-weighted image; T1W: T1-weighted image; DWI: diffusion weighted imaging; ADC: apparent diffusion coefficient

An MRI abdomen without contrast was done and showed an enlarged renal transplant in the right iliac fossa with the altered signal intensity of the renal parenchyma manifested by linear cortical and peri-pyramidal areas of high T2 (fluid) signal. There was an exophytic simple renal cortical cyst at the upper pole of the transplant measuring 5 cm, septated renal sinus spaces, and extensive perinephric septated clear fluid collection. There was evidence of diffuse edema of the surrounding fat, along with edema of the adjacent abdominal wall muscles and subcutaneous fat in the anterior and right lateral lower abdomen. The enlarged renal transplant and surrounding changes were causing a mass effect on the urinary bladder, uterus, and ovaries, displacing them to the left side of the pelvis. The uterus and ovaries were unremarkable. Native kidneys were atrophic with multiple cortical cysts. These features were suggestive of intrarenal, parapelvic, and perirenal lymphangiectasia of the renal transplant (Figure 3).

**Figure 4:**
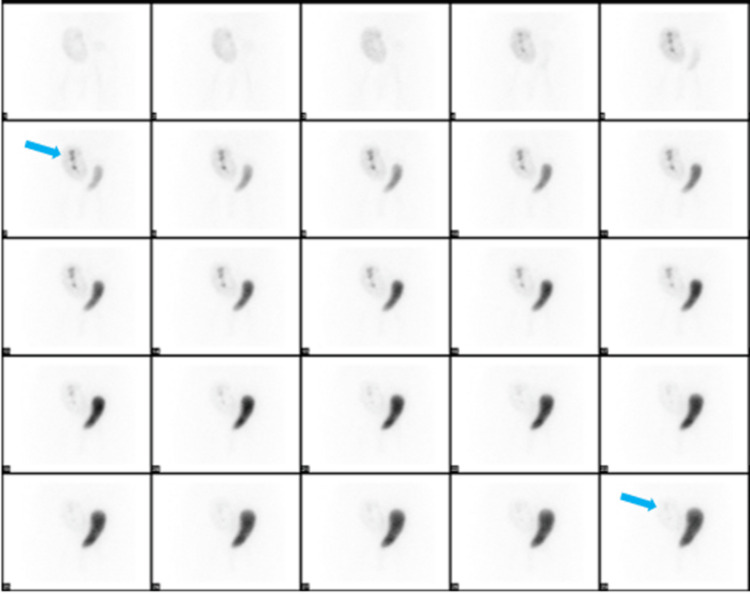
NM renogram demonstrating good function of the transplanted kidney (blue arrows) without obstruction or urine leakage.

A nuclear medicine (NM) renogram was done using Tc-99-MAG (mercaptoacetyltriglycine), which demonstrated good renal function in the transplanted kidney without any signs of obstruction or urine leakage (Figure 4).

She was started on oral mycophenolate mofetil 500 mg twice daily and prednisolone 5mg daily as a maintenance dose. The patient was doing well with conservative treatment and was discharged home with a follow-up in the renal transplant clinic.

## Discussion

The development of lymphatic vessels is controlled by pro-lymphangiogenic factors (vascular endothelial growth factors (VEGF) C and D) binding to VEGF3 receptors. Surgical complications are not uncommon after kidney transplantation. Lymphatic disorders are frequent, especially lymphocele, lymphorrhea, or lymphorrhagia, which most often occur in the first few months after transplantation and affect up to 40% of kidney transplant recipients. Renal lymphangiectasia, also known as renal lymphangiomatosis, is an uncommon complication after kidney transplantation [4]. Renal lymphatic capillaries are predominantly distributed around the interlobular and arcuate arteries of the cortex and drain into hilar collector vessels, while the medulla is generally devoid of lymphatics [5].

Renal lymphangiectasia is an uncommon benign renal abnormality. Hamroun et al. reviewed 50 reported cases; nearly all of them were detected in the native kidneys [4]. This may result in lymphatic fluid accumulation and distention of the lymphatics, leading to the formation of intra-parenchymal and perinephric fluid collections, which is called renal lymphangiectasia [6]. Renal lymphangiectasia cases account for only 1% of all the lymphatic abnormalities that have been reported in the literature [7]. To date, there have been two reported cases of extrarenal and one intrarenal lymphangiectasia post-renal transplantation. We present a second case of intrarenal lymphangiectasia, which occurred six years after renal transplantation [8].

Renal lymphatic capillaries are predominantly distributed around the interlobular and arcuate arteries of the cortex and drain into hilar collector vessels, while the medulla is generally devoid of lymphatics. The misconnection can evolve due to variable factors, including congenital factors such as lymphatic malformations and acquired factors like inflammatory reactions, infections, tumors, or traumas [5].

Clinically, renal lymphangiectasia is nonspecific. Most of the symptomatic patients are manifested clinically by non-specific symptoms, including abdominal or flank pain, abdominal distention due to ascites, abdominal lumps, hematuria, proteinuria, and hypertension. A few cases may have been clinically diagnosed due to complicated presentations, such as renal vein thrombosis or renal insufficiency [9]. Consequently, renal lymphangiectasia must be considered as one of the differentials besides hydronephrosis, urinoma, lymphoma, and polycystic kidney disease [8]. Asymptomatic cases are incidentally diagnosed in some studies [7].

The diagnosis of renal lymphangectasia cannot depend on history, clinical features, or lab tests. Medical imaging has been well-regarded as an essential tool for diagnosis [6]. On ultrasound, the pathology is shown in the renal sinus or in the perinephric region as anechoic cystic lesions with or without internal septa, called peripelvic renal lymphangiectasia (RLM) and perinephric RLM, respectively. Cystic lesions are sometimes seen extending from the renal parenchyma to the renal sinus. On CT imaging, the cystic lesions in the renal sinus, the peripelvic, or perinephric regions show fluid attenuation. The attenuation value of the CT varies between complicated and uncomplicated cysts; it measures between 0 and 10 Hounsfield units (HU) in the uncomplicated ones, whereas it goes up to more than 60 HU in the complicated ones. In the excretory phase of contrast-enhanced CT, the cystic lesions do not opacify with excreted contrast, which is a crucial manifestation that differentiates RLM from the distended pelvicalyceal system. Rarely, RLM can cause fluid collections in the retroperitoneal region because of the distended lymphatic channels. If the fluid collections do not complicate hemorrhage, they will appear clear on different imaging methods. On MRI scans, the cystic lesions manifest as hypointense and hyperintense lesions on T1- and T2-weighted images, respectively. In the context of intraparenchymal lymphangiectasia, the kidney size will be enlarged in association with a change in the differentiation between the cortical and medullary regions. In cases of renal insufficiency, iodinated contrast materials should not be administered. Instead, MRI with magnetic resonance (MR) excretory urography is the alternate imaging method for assessment. The images of T1-weighted MR excretory urography can benefit by providing functional and anatomical information after the administration of intravenous gadolinium contrast agents. Moreover, the internal septa can be easily seen on an MRI. In our study, US, MRI, and NM modalities were used for diagnosis [7].

Joubert syndrome (JS) is an exceedingly rare autosomal recessive neurodevelopmental hereditary disorder. The statistical estimation of JS with renal anomalies is 25%-33%. Nephronophthisis (medullary cystic disease complex), autosomal recessive polycystic kidney disease, multicystic dysplastic kidney disease, and nephronophthisis are the most reported renal anomalies in Joubert syndrome in the literature; however, we did not find any correlation between Joubert syndrome and lymphangiectasia [9].

Conservative medical management is the usual treatment in asymptomatic cases, but because renal failure and hypertension are possible complications in association with RLM, frequent follow-up is needed. Symptomatic cases of the least severity can undergo percutaneous aspiration of the fluid collection or surgical peritoneal fenestration (6). Whereas complicated cases and those with complaints of numerous recurrences may undergo laparoscopic ablation or nephrectomy. However, nephrectomy is not preferred currently because, in cases of asymmetrical bilateral abnormalities, the size of the contralateral renal cysts might increase [7]. Our patient received conservative medical management.

## Conclusions

Renal lymphangiectasia is an uncommon pathology, particularly in the case of kidney transplantation. It should be ruled out as one of the differential diagnoses for renal transplant patients who present with nonspecific clinical manifestations. The radiologists need to be familiar with the different imaging features, like renal subcapsular cystic lesions, which will aid in deciding the ideal therapeutic method for each patient and avoid needless invasive procedures for benign cases.
